# Self-assessed competence and need for further training among registered nurses in somatic hospital wards in Sweden: a cross-sectional survey

**DOI:** 10.1186/s12912-020-00466-2

**Published:** 2020-08-03

**Authors:** Renée Allvin, Birgitta Bisholt, Karin Blomberg, Carina Bååth, Sigrid Wangensteen

**Affiliations:** 1 Clinical Skills Center, Örebro University Hospital, S-701 85 Örebro, Sweden; 2 Faculty of Medicine and Health, School of Health Sciences, Örebro University, S-702 81 Örebro, Sweden; 3 Department of Health Science, Faculty of Health, Science and Technology, Karlstad University, S-651 88 Karlstad, Sweden; 4 Department of Health care Sciences, Ersta Sköndal Bräcke University College, S-100 61 Stockholm, Sweden; 5 Faculty of Health and Welfare, Östfold University College Fredrikstad, N-1757 Halden, Norway; 6 Department of Health Sciences in Gjøvik, NTNU, Norwegian University of Science and Technology, Trondheim, Norway

**Keywords:** Clinical competence, Nurse competence, Registered nurses, Self-assessment

## Abstract

**Background:**

Professional competence and continuous professional development is essential for ensuring high quality and safe nursing care, and it might be important for motivating nurses to stay in the profession. Thus, there is a need to identify the developmental process of nursing competency. Assessment of competence and need for further training helps to identify areas for quality improvement, and to design interventions in order to facilitate continuous competence development in different work contexts. The current study aimed to 1) describe registered nurses’ self-assessment of clinical competence as well as the need for further training, and 2) explore possible differences between registered nurses with varying lengths of professional experience as a nurse (≤ 0,5 year, > 0,5–5 years, and ≥ 6 years).

**Methods:**

A cross-sectional survey design was applied, using the Professional Nurse Self-Assessment Scale of clinical core competencies II. Registered nurses (*n* = 266) working in medical and surgical contexts in hospitals in Sweden responded (response rate 51%). Independent student t-test and analysis of variance were carried out.

**Results:**

Registered nurses assessed their competence highest in statements related to cooperation with other health professionals; taking full responsibility for own activities; and acting ethically when caring for patients. They assessed their need for further training most for statements related to assessing patients’ health needs by telephone; giving health promotion advice and recommendations to patients by telephone; as well as improving a creative learning environment for staff at the workplace. For self-assessed competence and need for further training, differences between the groups for 35 and 46 items respectively, out of 50 were statistically significant.

**Conclusions:**

Although the registered nurses assessed their competence high for important competence components expected of professionals such as cooperation with other healthcare professionals, it is problematic that knowledge of interactions and side-effects of different types of medication were reported as having the highest need of training. Longitudinal follow up of newly graduated nurses regarding their continuous development of competence as well as further training is needed.

## Background

Registered nurses (RNs) have a crucial impact on patient care. Professional competence and a continuous competence development are essential for ensuring high quality and safe nursing care [1–5]. Educational qualifications of nurses and patient to nurse staffing ratios have been directly linked to variations in hospital mortality [6]. The increasing number of reported cases of adverse events as health-related infections, pressure ulcers and fall injuries of a milder character during recent years [5] indicate a care that is not optimal.

Nursing competence is a core ability that is required for fulfilling nursing responsibilities [7], that is based by a formal education, which in Sweden corresponds to a three-year nursing program that leads to both a professional degree and a Bachelor’ degree. Although employers have to promote and motivate a continuous professional development [8], RNs have their own responsibility for maintaining their competence through continuous learning and use of judgement regarding their competence [9–11]. Formal education has to prepare students for a variety of healthcare settings, but job specific competencies should be obtained through the workplace and supported by employers [12].

According to Meretoja et al. (2004) competence is the ‘functional adequacy and capacity to integrate knowledge and skills to attitudes and values into specific contextual situations of practice’ (pp. 330–331) [13]. There is, however, inconsistency surrounding the definition of nurse competence, and the definitions have changed over time [14–17]. In order to find consensus, concept analyses identified clusters from the competence literature focusing on: discipline knowledge, discipline-specific skills, judgement, professional standards, interpersonal relationships, situational application of skills and knowledge, and outcome evaluation [2]. Differences in defining nurse competence may be a result of the various nurse roles, specialties and work contexts.

Different instruments to measure self-assessed nurse competence have been developed. These include the Nurse Competence Scale [13], the European Health Care Training and Accreditation Network Questionnaire Tool [18], the Nurse Professional Competence Scale [19], the Professional Nurse Self-Assessment Scale of clinical core competencies I (PROFFNurse SAS I) [20] and the PROFFNurse SAS II [21]. To our knowledge, the PROFFNurse SAS II is the only questionnaire measuring both self-assessment of competence and the need for further training.

Nursing has changed considerably and become a technology-enriched profession requiring such things as a willingness and motivation to incorporate digitalization into clinical practice [4]. Along with an increasing advanced and technical care, a requirement to perform more compassionate and fundamental care based on a person-centred approach has been emphasized [22]. RNs in a Swedish study described that a combination of different kinds of knowledge is important to provide good quality care [23]. In parallel with increasing demands on the nursing profession, nursing turnover continues to be a concern. The intention to leave the profession is most common among newly graduated nurses, and previous research has paid attention to nurses in the early stages of their career [24, 25]. In a Swedish longitudinal study every fifth nurse strongly intended to leave the profession after 5 years of employment [26]. During the same period, the proportion who were actively applying for jobs outside the profession more than doubled [26]. Leaving the profession generates a permanent loss of resources and an unstable nurse staffing that may compromise patient care [27], i.e. affect patient safety and possibility to provide a good quality care [6, 28, 29]. The fact that nurses leave the organization has negative consequences for both the individual and society. Due to nursing turnovers, there is a growing need to identify the developmental process of nursing competency [30]. A continued competence development might motivate nurses to stay in the profession [31].

To summarize, nurse turnover is an issue of concern in Sweden [26], as in other countries. This, together with a working environment where RNs experience higher stress, burnout and decreasing job satisfaction, makes it vital to further explore RNs’ role and competence. Assessment of competence and the need for further training helps determine professional development needs and areas for quality improvement [21]. Knowledge of RNs’ competence and needs for further training are fundamental to design interventions in order to facilitate continuous competence development in different work contexts. Therefore, the aim of the current study was to 1) describe registered nurses’ self-assessment of clinical competence and need for further training, and 2) explore possible differences between registered nurses with varying lengths of professional experience as a nurse.

Three research questions were identified:

1. How do RNs assess their competence?
2. How do RNs assess their need for further training?
3. Are there any differences between RNs with varying lengths of professional experience with respect to their
  - Self-assessment of competence?
  - Self-assessment of the need for further training?

## Methods

A cross-sectional survey design was used.

### Sample and setting

A convenience sample of RNs working in medical and surgical contexts in four hospitals in Sweden (two district hospitals, one county hospital, and one university hospital) was invited to participate. The RNs at the different departments had varying lengths of work experience as an RN, and different educational levels. All the RNs at the departments were given information about the study and were asked to participate. A total number of 528 RNs were invited to participate and 266 responded (response rate 51%).

### Data collection

The head nurses of the included departments, a director of studies, and a coordinator at the Clinical Skill Centre (CSL) at one of the hospitals, handed out a questionnaire to all the RNs. The RNs could return the questionnaires in boxes at the department or give it to the head nurse or the CSL coordinator. The survey was anonymous, no coding system to identify respondents was used. No reminder was given. Data were collected during September 2016 to February 2019.

### Questionnaire

The PROFFNurse SAS II) [21] was used for data collection in the present study. The theoretical foundation of PROFFNurse SAS I [20] and the PROFFNurse SAS II is Aristoteles’ three dimensions of knowledge (episteme, techê and phronesis). The development process of the PROFFNurse SAS I and II are described in Finnbakk et al. (2015) (version I) [20] and Wangensteen et al. (2018) (version II) [21]. The PROFFNurse SAS II used in the present study was developed ahead of a study among postgraduate nurses [21] and has also been used in studies by Taylor et al. 2020 [32], and Willmann et al. (2020) [33]. The questionnaire consists of 50 items and asks for responses on self-assessment of: a) competence (A-scale) and b) need for further training (B-scale). Both scales range from 1 to 10 where 1 indicates a very low level and 10 a very high level. Cronbach’s alpha values for the questionnaire for total score is reported to be 0.963 [21] and 0.936 [32]. In the present study the total Cronbach’s alpha values were 0.97.

### Data analysis

All data were entered and analysed in IBM SPSS Statistics 25. Frequency, mean, median, range and standard deviation were used to summarize the data. To analyse differences between RNs with varying lengths of professional experience, the sample was divided into three groups based on years as a nurse (≤ 0.5 year, > 0.5–5 years, and ≥ 6 years). The demarcation between groups was based upon national reports on RNs intent on leaving the profession within the first year [12]. Differences between the groups were tested by analysis of variance (ANOVA). Significant differences were further analysed with post hoc Tukey test. The significant level was set to < 0.05.

### Ethical approval

The study was carried out in accordance with the Declaration of Helsinki [34]. The participants received both verbal and written information about the aim, the procedure, and that participation was voluntary. Informed consent was sought by a covering letter explaining the purpose of the study. The voluntary act of returning the questionnaires was regarded as consent to participate. The study was approved by the Research Ethics Review Board of Uppsala University, Sweden (reg. no. 2011/071).

## Results

The respondents were between 22 and 67 years old (mean 33), including 234 (88%) females and 32 (12%) males with a mean experience as an RN of 6 years (0–44) (Table 1). All respondents worked in hospital somatic medical and surgical contexts. The total mean score for self-assessed competence (A-scale) was 7.59 (median 8, range 8) of a possible 10 points, and the total mean score for the need for further training (B-scale) was 5.6 (median 6, range 9) of a possible 10 points.

**Table 1:** Demographic variables (*n* = 266)

	N (%)	Mean (SD)	Median (min-max)
Female	234 (88)		
Male	32 (12)		
Age, years		33.3 (11.5)	28 (22–67)
Educational level			
Registered nurse	235 (88)		
Specialist nurse	31 (12)		
Years as RN		6 (10.3)	0.75 (0–44)
≤ 0.5 year	129 (48.5)		
> 0.5–5 years	61 (22.5)		
≥ 6 years	76 (29)		

### Self-assessed competence

The RNs assessed their competence highest in statements related to cooperation with other health professionals, taking full responsibility for own activities, and acting ethically when caring for patients (Table 2). The respondents assessed their competence lowest for statements related to assessing patients’ health needs by telephone (mean 4.50), giving health promotion advice and recommendations to patients by telephone (mean 4.73), and improving a creative learning environment for staff at the workplace (mean 5.58).

**Table 2:** Self-assessment of competence (A scale) = top 10 items

	Item	Content	Mean	SD
1	37	I consult other professional experts when required	9.37	1.066
2	32	**I take full responsibility for my own actions**	9.24**	1.222
3	24	I act ethically when caring for patients	9.05	1.069
4	28	I maintain an ethical approach towards my colleagues	8.98	1.117
5	39	I am cognisant of when my medical knowledge is insufficient when assessing patients’ health conditions	8.95	1.365
6	23	I take patients’ physical health needs (illness, pain, disabilities, etc.) into account when assessing and planning for the health and life situation of patients	8.83	1.185
7	38	I cooperate actively with other health professionals when coordinating patients’ nursing, care and treatment	8.62	1.493
8	34	**I understand the consequences my decisions may have for patients**	8.61*	1.479
9	10	**I utilise medical equipment in an appropriate and accurate manner**	8.53*	1.404
10	29	**I take active responsibility for creating a good working environment**	8.42*	1.730

Items in bold – significant differences between length of professional experience (ANOVA)

*p < 0.05

** p < 0.01

Separating the sample in RNs with a) ≤ 0.5 year (Group A), b) > 0.5–5 years (Group B), and c) ≥ 6 years (Group C) experience, the analyses of variance demonstrated statistically significant differences in self-assessed competence (A-scale) for 35 out of the 50 items (Table 3). The post hoc test of these 35 items demonstrated statistically significant differences between all three groups for four items (statements related to medication and treatment, quality development and routine improvement), between group A/C and B/C, for 14 items, between group A/B and A/C for one item, and between group A/C for 16 items (Table 3). RNs with ≥6 years of experience assessed their competence higher than group A and B for all 35 items. RNs with > 0.5–5 years’ experience assessed their competence higher than RNs in group A in five items (statements related to medication and treatment, quality development, routine improvement and making own decisions). The level of self-assessed competence increased with increased years of experience.

**Table 3:** Self-assessment of competence (A-scale). Statistical significant differences between groups based on experience as a nurse. ANOVA and post hoc Tukey

Item		Significant differences between groups	Mean difference	***p***
1	I am independently responsible for health assessment (systematic physical examination), examinations and treatment of patients with complicated medical conditions ^+^	+++	A/C: − 1.447 B/C: − 1.011	A/C: .000 B/C: .001
2	I am independently responsible for health assessment (systematic physical examination), examinations and treatment of patients with uncomplicated medical conditions	+++	A/C:-1.146 B/C: −.667	A/C: .000 B/C: .024
3	I plan and prioritise nursing and medical interventions	+	A/C: −.633	A/C: .005
4	I identify patients’ health problems	+++	A/C: −1.110 B/C: −.849	A/C: .000 B/C: .004
5	I assess patients’ symptoms	+++	A/C: −.904 B/C: −.586	A/C: .000 B/C: .034
6	I evaluate and modify patients’ medical treatment	+++	A/C: −1.127 B/C: −.670	A/C: .000 B/C: .031
7	I exclude differential diagnoses when assessing patients’ health conditions	+++	A/C: −1.318 B/C: −.914	A/C: .000 B/C: .012
8	I interpret, analyse and reach alternative conclusions about patients’ health conditions after a detailed mapping of health history and health assessment (physical examination)	+++	A/C: −.979 B/C: −1.030	A/C: .000 B/C: .001
9	I apply both subjective and objective methods when examining, treating and caring for patients	+	A/C: −.981	A/C: .000
10	I utilise medical equipment in an appropriate and accurate manner	+	A/C: −.648	A/C: .004
11	I have knowledge of the effects of medication and treatment for the patients I am responsible for	++++	A/B: −.905 B/C: −.805 A/C: −1.711	A/B: .003 B/C: .022 A/C: .000
12	I identify changes in patients’ health and medical conditions	+++	A/C: −.969 B/C: −.729	A/C: .000 B/C: .013
14	I systematically gather information from each patient about her/his health resources	+	A/C: −.668	A/C: .017
15	I have knowledge of the interactions of various types of medication and what side-effects they may cause for the patients I am responsible for	++++	A/B: −1.347 B/C: −.874 A/C: −2.221	A/B: .000 B/C: .036 A/C: .000
16	I generate a creative learning environment for staff at my workplace	+++	A/C: −1.775 B/C: − 1.129	A/C: .000 B/C: .015
17	I participate in quality development at my workplace	++++	A/B: −1.552 B/C: − 1.236 A/C: − 2.788	A/B: .000 B/C: .012 A/C: .000
18	I take responsibility for competence development at my workplace	+	A/C: −.700	A/C: .016
19	I improve routines/systems that fail to meet the needs of patients at my workplace	++++	A/B: −1.414 B/C: − 1.057 A/C: −2.471	A/B: .000 B/C: .027 A/C: .000
20	I am actively responsible for my own professional development	+	A/C: −.785	A/C: .009
27	I support and guide patients in mastering their illnesses and health problems	+	A/C: −.597	A/C: .035
29	I take active responsibility for creating a good working environment	+	A/C: −.803	A/C: .004
31	I make my own decisions in my work	++	A/B: −.646 A/C: −1.203	A/B: .032 A/C: .000
32	I take full responsibility for my own actions	+	A/C: −.705	A/C: .000
33	I am correct and accurate in speech and writing	+++	A/C: −1.099 B/C: −.851	A/C: .000 B/C: .012
34	I understand the consequences my decisions may have for patients	+	A/C: −.577	A/C: .019
35	I experience a division of responsibility between the physician and me as a nurse	+++	A/C: −1.211 B/C: − 1.221	A/C: .000 B/C: .003
36	I cooperate well with the physician	+++	A/C: −.715 B/C: −.824	A/C: .010 B/C: .013
40	I document the steps taken in assessing patients’ needs for nursing, care and treatment	+	A/C: −.746	A/C: .001
41	I reflect on my actions	+	A/C: −.633	A/C: .005
43	I perceive opportunities and have visions for how nursing and clinical paths for patients can be developed	+	A/C: −1.016	A/C: .002
44	I have a vision of how nursing should be developed at my workplace	+	A/C: −1.234	A/C: .000
45	I assess patients’ health needs by telephone	+++	A/C: −1.815 B/C: − 1.651	A/C: .000 B/C: .003
46	I give health promotion advice and recommendations to patients by telephone	+++	A/C: −2.103 B/C: −1.964	A/C: .000 B/C: .000
47	I give health promotion and illness preventive recommendations in accordance with national guidelines to patients	+	A/C: −1.153	A/C: .004
50	I report all incidents in accordance with the actual patient safety system	+	A/C: −1.873	A/C: .000

Groups based on experience as a nurse: Group A: ≤ 0.5 years, Group B: > 0.5–5 years, Group C: ≥ 6 years

^+^Significant differences between group A/C

^++^Significant differences between group A/B and A/C

^+++^Significant differences between group A/C and group B/C

^++++^Significant differences between all groups (i.e. A/C, A/B, B/C)

### Need for further training

Regarding the need for further training, the highest mean scores were found for statements related to medications and side-effects; health promotion and illness and preventive recommendations to patients; and giving health promotion advice to patients by telephone (Table 4). The lowest mean scores were found in statements related to cooperation with other health professionals (mean 4.02), acting ethically when caring for patients (mean 4.18), and taking full responsibility for own activities (mean 4.25).

**Table 4:** Self-assessment of need for further training (B scale) = top 10 items (i.e. competence needed most)

	Item	Content	Mean	SD
1	15	**I have knowledge of the interactions of various types of medication and what side-effects they may cause for the patients I am responsible for**	7.29**	2.336
2	47	**I give health promotion and illness preventive recommendations in accordance with national guidelines to patients**	6.83*	2.496
3	46	**I give health promotion advice and recommendations to patients by telephone**	6.76**	2.782
4	7	**I exclude differential diagnoses when assessing patients’ health conditions**	6.73**	2.342
5	11	**I have knowledge of the effects of medication and treatment for the patients I am responsible for**	6.69**	2.620
6	45	**I assess patients’ health needs by telephone, e-mail or other electronic devices**	6.64**	2.856
7	8	**I interpret, analyse and reach alternative conclusions about patients’ health conditions after a detailed mapping of health history and health assessment (physical examination)**	6.54**	2.277
8	19	**I improve routines/systems that fail to meet the needs of patients at my workplace**	6.43**	2.486
9	1	**I am independently responsible for health assessment (systematic physical examination), examinations and treatment of patients with complicated medical conditions**	6.34**	2.390
10	16	**I generate a creative learning environment for staff at my workplace**	6.33**	2.593

Items in bold – significant differences between length of professional experience (ANOVA)

*p < 0.05

** p < 0.01

Separating the sample in RNs with a) ≤ 0.5 year (Group A), b) > 0.5–5 years (Group B), and c) ≥ 6 years (Group C) experience, the analyses of variance demonstrated statistically significant differences for self-assessed need for further training for 46 out of the 50 items. The two items where no significant differences were found either for the A-scale or B-scale contains statements related to having an ethical approach. The post hoc test of the 46 items demonstrated statistically significant differences between all three groups for six items (statements related to health assessment, medical treatment, examinations, differential diagnoses, and incident reports), between group A/C and B/C for nine items, between group A/B and A/C for 19 items; and between group A/C for 12 items (Table 5). RNs with ≥6 years of experience assessed their needs for further training lower than group A and B for all 46 items. RNs with > 0.5–5 years experience assessed their needs for further training lower than RNs in group A in 25 items (Table 5). The self-assessed need for further training decreased with increased years of experience.

**Table 5:** Need for further learning (B-scale). Statistical significant differences between groups based on experience as a nurse. ANOVA and post hoc Tukey

Item		Significant differences between groups	Mean difference	***P***
1	I am independently responsible for health assessment (systematic physical examination), examinations and treatment of patients with complicated medical conditions	+++	A/C: 1.715 B/C: 1.109	A/C: .000 B/C: .016
2	I am independently responsible for health assessment (systematic physical examination), examinations and treatment of patients with uncomplicated medical conditions	++++	A/B: 1.063 B/C: 1.023 A/C: 2.086	A/B: .013 B/C: .039 A/C: .000
3	I plan and prioritise nursing and medical interventions	++	A/B: 1.411 A/C: 1.984	A/B: .001 A/C: .000
4	I identify patients’ health problems	++	A/B: 1.390 A/C: 2.161	A/B: .001 A/C: .000
5	I assess patients’ symptoms	++	A/B: 1.311 A/C: 2.056	A/C: .002 B/C: .000
6	I evaluate and modify patients’ medical treatment	++++	A/B: 1.107 B/C: 1.052 A/C: 2.159	A/B: .012 B/C: .038 A/C: .000
7	I exclude differential diagnoses when assessing patients’ health conditions	++++	A/B: .810 B/C: 1.216 A/C: 2.026	A/B: .049 B/C: .005 A/C: .000
8	I interpret, analyse and reach alternative conclusions about patients’ health conditions after a detailed mapping of health history and health assessment (physical examination)	+++	A/C: 1.593 B/C: 1.016	A/C: .000 B/C: .022
9	I apply both subjective and objective methods when examining, treating and caring for patients	++++	A/B: .996 B/C: 1.024 A/C: 2.020	A/B: .013 B/C: .023 A/C: .000
10	I utilise medical equipment in an appropriate and accurate manner	+	A/C: 1.115	A/C: .014
11	I have knowledge of the effects of medication and treatment for the patients I am responsible for	++	A/B: 1.613 A/C: 2.249	A/B: .000 A/C: .000
12	I identify changes in patients’ health and medical conditions	++++	A/B: .944 B/C: 1.228 A/C: 2.172	A/B: .029 B/C: .008 A/C: .000
13	I develop and administer health-promoting and illness-preventive actions for patients	+++	A/C: 1.811 B/C: .993	A/C: .000 B/C: .046
14	I systematically gather information from each patient about her/his health resources	++	A/B: 1.266 A/C: 1.707	A/B: .002 A/C: .000
15	I have knowledge of the interactions of various types of medication and what side-effects they may cause for the patients I am responsible for	++	A/B: 1.466 A/C: 2.045	A/B: .000 A/C: .000
16	I generate a creative learning environment for staff at my workplace	+	A/C: 1.542	A/C: .000
17	I participate in quality development at my workplace	+	A/C: 1.408	A/C: .001
19	I improve routines/systems that fail to meet the needs of patients at my workplace	+++	A/C: 1.794 B/C: 1.187	A/C: .000 B/C: .015
21	I take patients’ mental health needs (mood swings, feelings of hopelessness, depression, etc.) into account when assessing and planning for the health and life situation of patients	++	A/B: 1.168 A/C: 1.454	A/B: .009 A/C: .000
22	I take patients’ spiritual health needs (feelings of meaninglessness, existential needs, beliefs, fear of death, etc.) into account when assessing and planning for the health and life situation of patients	++	A/B: 1.007 A/C: 1.634	A/B: .036 A/C: .000
23	I take patients’ physical health needs (illness, pain, disabilities, etc.) into account when assessing and planning for the health and life situation of patients	++	A/B: 1.096 A/C: 1.012	A/B: .025 A/C: .029
25	I identify and assume responsibility for patients’ own health resources in planning nursing care	+	A/C: 1.149	A/C: .007
26	I take patients’ social health needs (leisure activities, friends, financial situation, etc.) into account when assessing and planning for the health and life situation of patients	+++	A/C: 1.927 B/C: 1.110	A/C: .000 B/C: .031
27	I support and guide patients in mastering their illnesses and health problems	+	A/C: 1.590	A/C: .000
29	I take active responsibility for creating a good working environment	+	A/C: 1.132	A/C: .014
30	I put emphasis on patients’ own wishes when assessing and planning for nursing care and medical treatment	++	A/B: 1.007 A/C: 1.380	A/B: .048 A/C: .002
31	I make my own decisions in my work	++	A/B: 1.359 A/C: 2.450	A/B: .006 A/C: .000
32	I take full responsibility for my own actions	++	A/B: 1.547 A/C: 2.137	A/B: .002 A/C: .000
33	I am correct and accurate in speech and writing	+	A/C: 2.111	A/C: .000
34	I understand the consequences my decisions may have for patients	++	A/B: 1.367 A/C: 2.322	A/B: .006 A/C: .000
35	I experience a division of responsibility between the physician and me as a nurse	+++	A/C: 2.503 B/C: 1.626	A/C: .000 B/C: .003
36	I cooperate well with the physician	+++	A/C: 1.948 B/C: 1.392	A/C: .000 B/C: .011
37	I consult other professional experts when required	++	A/B: 1.123 A/C: 1.504	A/B: .033 A/C: .001
38	I cooperate actively with other health professionals when coordinating patients’ nursing, care and treatment	++	A/B: 1.571 A/C: 1.681	A/B: .001 A/C: .000
39	I am cognisant of when my medical knowledge is insufficient when assessing patients’ health conditions	+	A/C: 1.420	A/C: .004
40	I document the steps taken in assessing patients’ needs for nursing, care and treatment	++	A/B: 1.468 A/C: 1.714	A/B: .003 A/C: .000
41	I reflect on my actions	++	A/B: 1.238 A/C: 1.681	A/B: .011 A/C: .000
42	I analyse and evaluate my work continuously	+	A/C: 1.676	A/C: .000
43	I perceive opportunities and have visions for how nursing and clinical paths for patients can be developed	+	A/C: 1.716	A/C: .000
44	I have a vision of how nursing should be developed at my workplace	+	A/C: 1.541	A/C: .000
45	I assess patients’ health needs by telephone	+++	A/C: 1.702 B/C: 1.657	A/C: .000 B/C: .004
46	I give health promotion advice and recommendations to patients by telephone	+++	A/C: 1.901 B/C: 1.989	A/C: .000 B/C: .000
47	I give health promotion and illness preventive recommendations in accordance with national guidelines to patients	+	A/C: 1.082	A/C: .015
48	I have a supportive ongoing dialogue with patients about their needs and wishes	++	A/B: 1.295 A/C: 1.869	A/B: .005 A/C: .000
49	I focus on relatives’ need for support and guidance	++	A/B: 1.045A/C: 1.989	A/B: .035 A/C: .000
50	I report all incidents in accordance with the actual patient safety system	++++	A/B: 1.681 B/C: 1.294 A/C: 2.975	A/B: .000 B/C: .020 A/C: .000

Groups based on experience as a nurse: Group A: ≤ 0.5 years, Group B: > 0.5–5 years, Group C: ≥ 6 years

^+^Significant differences between group A/C

^++^Significant differences between group A/B and A/C

^+++^Significant differences between group A/C and group B/C

^++++^Significant differences between all groups (i.e. A/C, A/B, B/C)

### Concurrence between self-assessed competence and need for further training

Seven of the top 10 items regarding highest need for further training (Table 4) were found among the ten items with lowest self-assessed competence. In the same way, seven of the top 10 items assessed with lowest need for further training were found among the ten items with highest self-assessed competence (Table 2). Statistically significant differences between group A (≤ 0.5 year) and group B (> 0.5–5 years) were seen in two items: “*I have knowledge of the interactions of various types of medication and what side-effects they may cause for the patients I am responsible for*” (item 15), and “*I make my own decisions in my work*” (item 31) in both the A-scale and the B-scale. Statistically significant differences between group B (> 0.5–5 years) and group C (≥ 6 years) were seen in 18 and 15 items for self-assessed competence and need for further training respectively. Eleven of these items were found in both the A-scale and the B-scale (1–2, 6–8, 12, 19, 25, 36, 45–46) (Tables 3 and 5). For most items there was no concurrence between self-assessed competence and the need for further training regarding statistically significant differences between the groups.

## Discussion

The aim of this study was to describe nurses’ self-assessment of clinical competence and need for further training. The items where the respondents assessed their competence highest related to statements of cooperating with other healthcare professionals and experts; taking full responsibility for own activities; and acting ethically when caring for patients. As previously stated, in a study exploring clinical competence and need for further training among RNs in postgraduate programmes in Europe [21], these are all components of phronesis, understood as practical wisdom [35], and fundamental competence components expected of professional nurses [9]. Personal responsibility implies a moral requirement to choose actions to take ethical responsibility for the patient [36].

The lowest self-assessments for competence were seen for statements related to managing healthcare without seeing the patient (i.e. using the telephone, e-mail or other electronic devices), and giving health promotion advice. It might be argued that the RNs in this study worked with hospitalized patients, and therefore did not have the same experience of assessing health needs by telephone, as if they had been working in primary care. However, nursing is becoming a technology-enriched profession [4], with an increasing use of information- and communication technologies in healthcare, i.e. e-Health [37], irrespective of healthcare contexts. Both managing healthcare without seeing the patient and giving illness and preventive recommendations were among the items the respondents assessed highest regarding the need for further training, which indicate that they are aware of their flaws.

*The respondents also assessed their competence low* regarding improving a creative learning environment for staff at the workplace, improving routines, and knowledge of interactions and side effects of various types of medication. This can imply a challenge because establishing key abilities, such as identifying a learning need contributes to improving nursing practice [38]. Positive work experiences in the first year of practice, in terms of sharing experiences and getting encouraging support from colleagues, has been pointed out as important for remaining motivated at work [39], and for sustaining the future of the profession [40]. These findings are of importance to highlight in connection with the development of both nursing programmes and of introduction programmes for newly graduated RNs.

The second aim of the study was to explore if there were differences with respect to self-assessment of clinical competence and the need for further training between RNs with varying lengths of professional experience as a nurse. Statistically significant differences between the RN groups were seen for several items regarding both self-assessed competence and self-assessed need for further training. Previous research has demonstrated that nurse competence differs depending on length of work experience [30, 41], and frequency of using these experiences [42], which has been found to explain up to 40% of variance in self-assessed competence among newly graduated nurses [43]. Furthermore, higher academic degree has been connected to higher self-assessed competence among RNs in postgraduate programmes [21], and among operating theatre nurses [44]. According to Aiken et al. (2014), RNs with an academic degree are associated with improved patient outcomes [6]. Although the educational environments are academic, there is a risk that newly graduated nurses will be introduced into a vocational and task-oriented view of the profession if employers do not take responsibility for the academic culture within the healthcare sector, which could jeopardize a safe nursing care [12, 45]. RNs have an important coordination position in patient safety issues and work in close proximity to the patients where decisions of importance for patient safety are made [28, 46].

In the present study only four of the top 10-items for self-assessed competence showed statistically significant differences between the groups. For example, no statistically significant differences were found between the groups regarding two of the items with highest competence assessment: cooperating with other healthcare professionals and experts (item 37) and acting ethically (item 24). This finding could be seen in relation to interprofessional collaboration, which has become an important component of a well-functioning healthcare system [47]. A previous study exploring interprofessional collaboration between nurses and junior doctors showed that nurses needed to be more active by taking more responsibility in improving their ability to collaborate with other professionals [48]. However, according to Regan et al. (2015) nurses are more confident in interprofessional collaboration when they control their work situation and have the independence to make patient care decisions on their own [49]. This might explain why the RNs in the present study, regardless of experience, assessed their competence as high.

### Methodological considerations

As with any questionnaire-based study, some issues regarding the sample and response rate of the study should be noted [50]. The convenience sample of RNs, in the present study, may be associated bias at the specific data collection day, such as staff turnover, sick leave, and changing in the working schedules. The response rate was 51%, which could be considered less than ideal. However, no reminder was given, and the questionnaire was distributed to the RNs to be completed, without setting a strict timeframe. The response rate might have been higher if a follow-up reminder had been used. However, it should be noted that low response rates do not mean that the results are biased [51]. High workload and a comprehensive questionnaire were stated as reasons for not answering the questionnaire. Even if the validity/reliability of self-assesed measures has been questioned, it has been reported as the most common form of competence assessment [52]. In the present study, the correspondence between the respondents’ self-assessment of competence and the perceived need for further training indicates that their self-assessment of competence may be reliable. The timeframe for how long an RN is considered to be newly graduated is undetermined as the transition and experience are individual [39]. The decision to limit one of the groups to 6 months of experience as an RN, instead of 1 year that is commonly done, was based on the reports of turnover early in the nursing career [12, 45].

## Conclusions

A sample of hospital-based nurses in Sweden self-reported their competence as high (80%, a median score of 8 of a possible 10 points) but ranked the need for further education/training as lower overall (median 6 or 60%). Although the RNs assessed their competence high for important competence components expected of professionals such as cooperation with other healthcare professionals, it is problematic that knowledge of interactions and side-effects of different types of medication were reported as having the highest need of training. Longitudinal follow up of newly graduated RNs regarding their continuous development of competence as well as further training is needed.

## Data Availability

The dataset analysed during the current study are available from the corresponding author on reasonable request.

## Abbreviations

RN: Registered Nurse
PROFFNurse SAS: Professional Nurse Self-Assessment Scale
CSL: Clinical Skill Centre
